# Occupational Therapists’ Views on Using a Virtual Reality Interior Design Application Within the Pre-Discharge Home Visit Process

**DOI:** 10.2196/jmir.3723

**Published:** 2014-12-18

**Authors:** Anita Atwal, Arthur Money, Michele Harvey

**Affiliations:** ^1^Brunel UniversitySchool of Health Sciences and Social CareBrunel UniversityLondonUnited Kingdom; ^2^Brunel UniversityDepartment of Computer ScienceBrunel UniversityLondonUnited Kingdom

**Keywords:** occupational therapy, pre-discharge home visits, virtual reality, 3D, patient collaboration, patient engagement, empowerment, technology assisted care, patient perceptions

## Abstract

**Background:**

A key role of Occupational Therapists (OTs) is to carry out pre-discharge home visits (PHV) and propose appropriate adaptations to the home environment in order to enable patients to function independently after hospital discharge. However, research shows that more than 50% of specialist equipment installed as part of home adaptations is not used by patients. A key reason for this is that decisions about home adaptations are often made without adequate collaboration and consultation with the patient. Consequently, there is an urgent need to seek out new and innovative uses of technology to facilitate patient/practitioner collaboration, engagement, and shared decision making in the PHV process. Virtual reality interior design applications (VRIDAs) primarily allow users to simulate the home environment and visualize changes prior to implementing them. Customized VRIDAs, which also model specialist occupational therapy equipment, could become a valuable tool to facilitate improved patient/practitioner collaboration, if developed effectively and integrated into the PHV process.

**Objective:**

The intent of the study was to explore the perceptions of OTs with regard to using VRIDAs as an assistive tool within the PHV process.

**Methods:**

Task-oriented interactive usability sessions, utilizing the think-aloud protocol and subsequent semi-structured interviews were carried out with seven OTs who possessed significant experience across a range of clinical settings. Template analysis was carried out on the think-aloud and interview data. Analysis was both inductive and driven by theory, centering around the parameters that impact upon the acceptance, adoption, and use of this technology in practice as indicated by the Technology Acceptance Model (TAM).

**Results:**

OTs’ perceptions were identified relating to three core themes: (1) perceived usefulness (PU), (2) perceived ease of use (PEoU), and (3) actual use (AU). Regarding PU, OTs believed VRIDAs had promising potential to increase understanding, enrich communication and patient involvement, and improve patient/practitioner shared understanding. However, it was unlikely that VRIDAs would be suitable for use with cognitively impaired patients. For PEoU, all OTs were able to use the software and complete the tasks successfully; however, participants noted numerous specialist equipment items that could be added to the furniture library. AU perceptions were positive regarding use of the application across a range of clinical settings including children/young adults, long-term conditions, neurology, older adults, and social services. However, some “fine tuning” may be necessary if the application is to be optimally used in practice.

**Conclusions:**

Participants perceived the use of VRIDAs in practice would enhance levels of patient/practitioner collaboration and provide a much needed mechanism via which patients are empowered to become more equal partners in decisions made about their care. Further research is needed to explore patient perceptions of VRIDAs, to make necessary customizations accordingly, and to explore deployment of the application in a collaborative patient/practitioner-based context.

## Introduction

### Background

With an anticipated rise in the demand for health care resources as a result of an ageing population [1], government initiatives see innovations in technology for health care as one of the few areas in which there still remains capacity for reducing costs and improving quality of service for patients [2]. In particular, the recently proposed long term version for the National Health Service (NHS) under the banner of “Equity and Excellence: Liberating the NHS” [3] demonstrates the UK government’s commitment to innovation via the use of Information and Communication Technology (ICT). ICT is seen as a key lever in delivering person-centered, preventative, re-abling, and personalized care. If this vision is to be realized, it is crucial to “empower and liberate clinicians to innovate”’ [3], hence enabling practitioners to adopt and integrate new technologies and practices with a view to improving patient health outcomes. A central role of incorporating ICT into heath care delivery is to provide more effective patient–centred health care that creates opportunities for both practioners and patients to work collaboratively in the consultation and decision making processes. [4,5]. Enabling patient/practitioner collaboration will improve the extent to which the patient is aware of their health issues, consequently improving levels of patient engagement, adherence, and satisfaction [6]. Promoting innovative applications of technology for health care is seen as playing a central role in enabling patients to take responsibility for their own care, and improve and sustain quality of life by making it possible to live independently within their own homes for longer [7].

A primary area of focus within the domain of Occupational Therapy is to enable patients to live independently within their homes. In order to facilitate this, a key role of an occupational therapist (OT), across Europe, Australia, and North America [8], is to carry out a pre-discharge home visit (PHV) with the patient to facilitate appropriate, safe, and successful discharge from hospital to home [9]. The aim of the PHV is for the clinician to visit the home with the patient to provide additional information about how the patient will cope within their home environment after discharge. During the visit the OT may propose modifications to the home environment, where appropriate, to enable the patient to function at a satisfactory level of independence after discharge [10]. Modifications to the home environment may include installation of specialist assistive equipment such as bed hoists, support rails, shower seats, grab rails, and so forth. Furthermore, often patients may be faced with the prospect of using a wheelchair or walking frame to aid mobility, which may also dictate that alterations must be made to the layout of the home to accommodate access. Recent research in the field of occupational therapy has revealed that PHVs can be cumbersome, highly resource intensive, and sub-optimal for patients [8,11]. One significant issue is that decisions made regarding home adaptations are often made without adequate collaboration and consultation with the patient [10,12]. Currently, the only real opportunity to consult and collaborate with the patient is while the clinician and patient are together in the home as part of the PHV process. However, patients have reported that they find this anxiety-provoking and feel as if they are being tested/assessed in terms of their mobility around the home; hence, they do not feel able to collaborate as an equal partner for fear of not being discharged home after the visit [10]. This could influence use of assistive devises since more that 50% of home adaptations are not used after discharge home, resulting in sub-optimal health outcomes and significant wastage of resources [12]. This is perhaps no surprise when considering how personalized and sensitive the home setting is [13], Coupled with the fact that there is no readily available tool or technique that assists patients and practioners to jointly visualize and explore the home environment according to the patients’ personal needs [14].

There is a need to seek out and develop new and innovative uses of technology that enable patients and practitioners to jointly understand and visualize the complexities and meanings associated with the home environment, to envisage the challenges that are likely to be encountered within the home, and to collaborate and contribute equally to developing solutions to these challenges [15]. This is likely to lead to many positive health outcomes such as improved adherence, engagement, and patient satisfaction [6].

### Virtual Reality Interior Design Applications for Occupational Therapy

**Figure 1:**
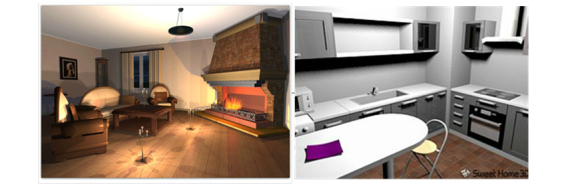
Examples of virtual home environments, lounge (left), kitchen (right), produced using Virtual Reality Interior Design Applications (VRIDA).

Over the past decade, Virtual Reality (VR) has become a valuable tool that has been applied to a range of health care scenarios [16]. The term VR typically relates to interactive three-dimensional (3D) computer-generated environments that simulate being present within the real world equivalent of that environment [17]. Application areas of VR span across a range of domains including interior design, health care, military and defense, education, and entertainment and gaming [18]. Specifically relating to health care, perhaps the most well-noted application of VR has been for the treatment of phobia such as public speaking anxiety [19] and the treatment of claustrophobia [20]. More specifically within the domain of home interior design, virtual reality interior design applications (VRIDA) serve as a valuable assistive tool for negotiating adaptations between designers and home owners [21]. VRIDA allows individuals to design or redesign their homes virtually, prior to making these changes a reality. The advantages of using VRIDA in interior design include improved collaboration between the home owner and the designer, and enhanced understanding and communication of design options. It also brings design misconceptions to the forefront of discussion, facilitating active participation by all parties involved, and aiding the process of achieving consensus between all parties [22,23]. Figure 1 provides some examples of 3D home environments produced using VRIDA. The specific VRIDA used to produce the example environments was “SweetHome 3D”, a freely available open-source 3D interior design software application [24].

In light of the need for improved collaboration between OTs and patients, this research proposes to explore the use of VRIDA to aid the PHV process and gain insights into patient and practitioner experiences of its application in practice. The prospect of using VRIDA has potential to respond to a number of the issues that currently limit the effectiveness of PHVs. VRIDA would serve as a tool that enables occupational therapists to rapidly create the 3D representation of the patient’s home, allowing the patient and practitioner to jointly visualize the interior of the home and trial a range of adaptations and specialist equipment within it. This would enhance collaboration between clinician and patient and assist them in making shared decisions about how this sensitive and personalized space may be best adapted specifically to the patient’s individual needs. It would also provide an interactive simulation of the home, enabling the patient to “walk” through the home, via a personal computer or laptop, which could help therapists to better consider barriers to everyday performance and enhance the patient’s insight and motivation to participate in tailored interventions. VRIDA would provide the patient with the valuable opportunity to consult as an expert on their own needs, and participate as an equal partner in decision making, without feeling as if their mobility is being assessed, as is often the case when visiting the home in person with the practitioner. To date, however, the gains that VRIDA could bring to occupational therapy practice are yet to be capitalized upon, as little research has been carried out within this particular health care context.

### Practitioner Perceptions and Technology Acceptance

The insight of practitioners is extremely valuable and should be employed at all stages of technology development and deployment. Research with health care practitioners has shown that they are more likely to adopt technologies if these are viewed as compatible with current practice [25]. Compatibility issues could relate to time, existence of evidence on positive outcomes in practice [26], organizational issues [27], and attitudes of professionals towards technology [28]. Therefore, the application of VRIDA must be accepted by therapists if it is to serve as a feasible tool that may be used within occupational therapy in practice [28]. If VR therapies are not perceived as usable or likable in actual clinical settings, it is unlikely that the technology will remain in use long enough for an evidence-base to be explored and established.

Over the past two decades, much research effort has been invested into understanding end users’ reactions and motivations to technology acceptance, adoption, and use [29]. Although a large portion of this research effort has been focused within the Information Systems research domain, more recently, there is increasing interest in gaining a better understanding of the factors that influence user acceptance, adoption, and use of technology within the health care domain [30]. To the best of our knowledge, there is no research yet that explores barriers to acceptance, adoption, and use of VRIDA technology for use within the occupational therapy context or its application to the PHV process. The Technology Acceptance Model (TAM) is perhaps the most notable theory applied in the explanation of user motivations, attitudes, and responses to acceptance and use of technology [31]. Despite its relative simplicity, even the most basic form of TAM is typically seen to provide an explanation of approximately 40% of issues related to technology acceptance [29].

TAM proposes that when presented with a new technology, users’ behavioral intention to use and their Actual Use (AU) of technology are typically mediated by two key factors: Perceived Usefulness (PU), which is the extent to which the user perceives that the new technology will aid them in performing the task at hand, and Perceived Ease of Use (PEOU), which is the extent to which the individual believes using the technology would be free of effort [32]. TAM is now increasingly being applied within the health care research domain [33]. Examples include exploring the acceptance of: telemedicine technology by nurses [34], Personal Digital Assistants (PDAs) by physicians [35], portable postural assessment technologies for use by physiotherapists [36], mobile picture archiving technologies for dental care [37], and a range of customizable and wearable health care devices for patients and practitioners [38]. Although the vast majority of TAM research to date has been quantitative, there is increasing recognition that qualitative enquiry, particularly in the early explorative stages, is well suited to scoping the design and development of new innovations and identifying the range of factors that may affect the acceptance, adoption, and use of a specific technology [39].

The aim of this study is to explore occupational therapists’ perceptions of VRIDA and to gain insights into the feasibility of using VRIDA as a tool to aid the PHV process in relation to the key factors outlined in the technology acceptance model. The next section provides details of the study carried out to achieve this aim. The results of this study are then presented, followed by a discussion of the implications of the findings in the context of existing research literature and outlining the study limitations. Finally, the study is concluded and future research directions are considered in light of the findings.

## Methods

### Overview

The aim of this study was to explore the perceptions of OTs relating to the three TAM factors (PU, PEOU, and AU) and the potential feasibility of using VRIDA applications as an assistive tool that may be used within the PHV process.

### Participants

A convenience sampling strategy was used for recruitment of participants for this study. The inclusion criteria were that participants were practicing OTs within the UK health sector and that they were familiar with using desktop computers and typical applications such as Microsoft Word and accessing email. Potential participants were primarily identified from the researchers’ existing social network contacts list (ie, LinkedIn contacts) and subsequently contacted by email in the first instance and invited to take part in this study. No financial incentives were offered to take part in the study, hence participation took place purely on a voluntary basis. A total of seven OTs were recruited and took part in the study. This number of participants is in excess of the recommended threshold of five participants typically required to carry out effective think-aloud interaction and usability testing [40,41]. Five of the participants were female and two were male. The amount of clinical experience that participants had ranged from 5-20 years and areas of specialty included community-based social services, older persons, mental health, acute care, and pediatrics. Table 1 provides a summary of the participant profiles.

**Table 1:** Summary of participant profiles.

Participant	Gender	Years practicing	Area of specialty
A	Male	More than 5 years	Social services (community)
B	Female	More than 10 years	Senior Therapist Older Person
C	Female	More than 5 years	Senior Therapist Older Person
D	Female	Less than 5 years	Social services (community)
E	Male	More than 20 years	Mental Health Team Leader
F	Female	More than 10 years	Senior Therapist Acute Care
G	Male	More than 5 years	Pediatrics

### Orientation Task Using a VRIDA

On arrival, information sheets were distributed to users prior to participation in the session, the content of which was worked through with each participant individually. The information sheet provided a brief background and context and purpose to the study, and summarized the main activities that would take place during the course of the session. Participants were encouraged to ask questions throughout the process, and any questions were answered as they arose. Participants were then asked to complete a consent form in which their ethical rights were explained in terms of informed consent, withdrawal, and anonymity.

**Figure 2:**
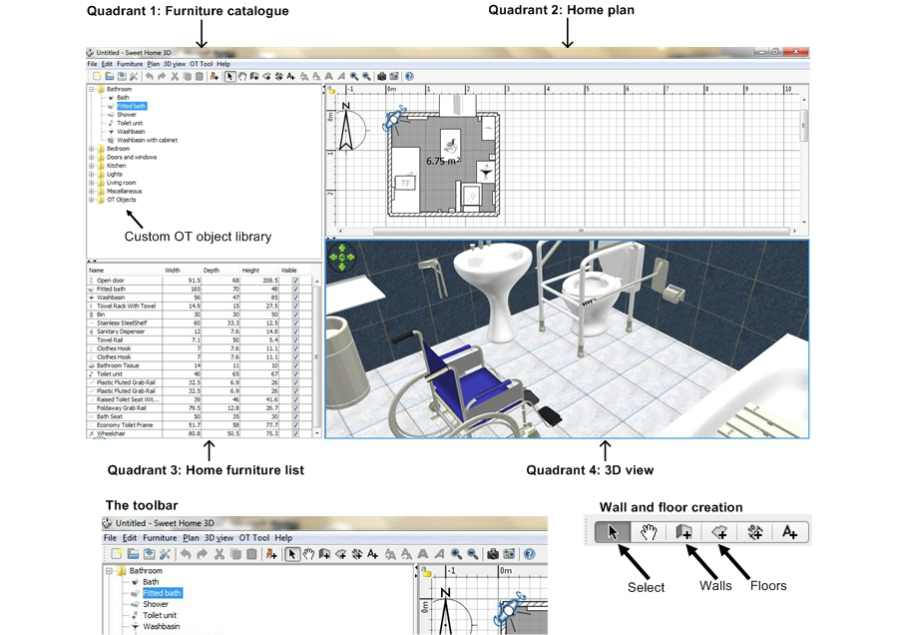
The customized SweetHome 3D application interface.

Participants were given the task of using a VRIDA to design the interior of a room that would typically represent a patient’s home environment. The VRIDA software application used for the purposes of this task was a customized version of SweetHome 3D [24]. Figure 2 illustrates the SweetHome 3D application interface used by participants to design and develop home interiors.

The SweetHome 3D application interface is made up of four main functional quadrants: (1) furniture catalogue, (2) home plan, (3) home furniture list, and (4) 3D view. For the purposes of this study, the application has been customized to include a library of specialist OT assistive equipment necessary for OTs to make typical home adaptation recommendations as part of the PHV process. These artefacts were presented within the furniture catalogue quadrant of the application in a folder entitled “OT Objects”. Occupational therapy assistive devices featured in the library included ramps, a range of grab rails, a bath hoist, a wheelchair, toilet frame, and seat. The custom OT objects library folder, how this was integrated into the furniture catalogue navigation pane, and examples of some of these OT objects (wheelchair and toilet frame) are presented in Figure 2, as well as how these OT objects may be modelled within an example 3D view of a bathroom environment.

Prior to the main task of designing a typical patient room of their choice, participants were provided with basic written instructions, presented in Table 2, outlining the key steps necessary to create a room using the application.

Printed screenshots of the SweetHome 3D interface and the 4-quadrant map of the software (similar to those presented in Figure 2) were also provided alongside the written instructions. Using the resources provided, participants were asked to design a bathroom in order to practice developing an environment prior to moving on to the main task. After the participants perceived they were confident in utilizing the software, they were asked to proceed with the main task.

**Table 2:** Written instructions for initial familiarization and orientation with SweetHome 3D.

Instructions	
**Create your room**	
	Draw floor in Quadrant 3 using the Floor button (follow instructions in pop-up box)
	Draw walls in Quadrant 3 using the Walls button (follow instructions in pop-up box)
**Furnish your room**	
	Choose objects from Quadrant 1 using the Select tool
	Drag and drop into Quadrant 3, arrange using the Select tool
	An inventory of these objects will appear in Quadrant 2
**Decorate your room**	
	Select a wall or floor in Quadrant 3, it will highlight in blue once it is selected
	Right-click on highlighted wall or floor, select “Modify Walls” or “Modify Floor”, choose colors/textures
**Visit your room**	
	Go to “3D View” menu at the top menu, choose “Virtual Visit”
	A figure will appear in Quadrant 3 and the view in Quadrant 4 will change
	Move and click in Quadrant 4 to look around the room
**Save your room**	
	Go to “File” at the top menu
	Name your file and save to the desktop

### Think-Aloud Interior Home Design Task

For the main task, participants were asked to design a room of their choice, which they believed represented a typical room within a patient’s home. They were also welcome to insert assistive equipment where they deemed necessary. Participants were asked to design the room of choice from scratch, while adopting a ‘think-aloud’ approach, which enabled them to verbally share their thoughts while interacting with the application [42]. Think-aloud is a valuable research technique that is typically used for the real-time capture of, not only client preferences and thoughts while interacting with software applications, but also the reasoning behind these preferences and thoughts. It is frequently used within usability testing and has been used within occupational therapy to explore clinical reasoning [43]. It was important that users did not feel pressured, as it was anticipated that this may impact on the level of think-aloud thoughts they felt comfortable sharing during the task. Therefore, users were reminded throughout the task that there was no urgency in completing the task, and encouraged to take as long as they required to provide comments and to interact with the SweetHome 3D application. During the sessions, standard prompts to think aloud, such as “what are you thinking?” and “what are you doing now?” were used whenever the researcher felt there were extended periods of silence. The use of task-focused prompts such as these ensured that participants’ attention remained on the task at hand but also that sufficient data and commentary relating to their interactions with the software application were elicited [44]. All sessions were audio-recorded and the researcher also took written notes during the session. At the end of the session, a discussion was held with the user, giving them the opportunity to elaborate on any of the points they made during the session and to reflect on their experience of using the application and perceptions relating to its ease of use, usefulness, usability, and the feasibility of it actually being used as a tool to assist in the PHV process.

### Data Analysis

Template analysis was used to analyze the interview data. This is a form of thematic analysis, which involves development of a coding template that represents a summary of the themes that are seen by the researcher(s) as being of importance within the dataset [45]. The approach taken to this was both inductive, as some themes were closely linked to the data, and other themes were driven by theory and the researchers’ analytical interest [46]. Analysis often begins with some *a priori* themes/codes that are of interest to the researcher. In this case, the interview data was approached with the broad aim of exploring factors that are related to TAM. Hence, analysis considered the participant perceptions of the feasibility, usability, and utility of VRIDA for PHV interventions within the context of three high-level TAM themes relating to PU, PEOU, and AU. The approach taken in the analysis of this data is in line with what Madhill et al [47] refer to as the ‘contextual constructivist’ position. In this case, it is accepted that there are many interpretations that may be made of a given phenomenon, which depends upon the focus of the researcher and the context in which the research is carried out. Hence, the themes and sub-themes that emerge as a result of the analysis are partly a product of these factors.

As an initial step, all interview recordings were transcribed into text format. The textual dataset in its entirety was perused to conceptualize the data and its relationship to the *a priori* themes that existed at a high level. The entire dataset was closely read and patterns in the data were noted. Sequences of data that represented “the most basic segment, or element, of the raw data or information that can be assessed in a meaningful way regarding the phenomenon” [48] were identified and assigned a code name. The dataset was then examined iteratively through several stages of splicing, linking, deleting, and reassigning themes and sub-themes. In this way, a final representation of the themes in the data was produced. The themes did not ‘emerge’, because they did not have a concrete existence in the data, rather they were constructed as part of the interpretative work. The analysis goes beyond the surface meaning of the data and tries to “identify the underlying ideas, assumptions, and conceptualizations—and ideologies—that are theorized as shaping or informing the semantic content of the data” [46]. The first and second authors coded the data and discussed inconsistencies where these arose until a clear consensus of the main themes was reached. The main themes are those drawn from multiple contributions and that represent issues that are clearly central to the participants themselves. Within these themes, we have identified sub-themes that depict the breadth of positions that were adopted within the main themes. For a detailed description of the thematic template analysis process, see King [45], and similarly for the thematic analysis process, see Joffe and Yardley [49] and Silverman [50].

### Ethical Considerations

The study was reviewed and approved by the Brunel University Research Ethics Committee prior to any data collection. All participants taking part in the study were guaranteed confidentiality and anonymity. Signed consent forms were obtained from all participants prior to taking part in the semi-structured interviews. Participants were informed of their right to withdraw from the study at any time. This was done both in writing and verbally.

## Results

### Overview

**Figure 3:**
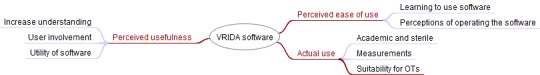
Thematic mind map of themes and sub-themes.

The results of the analysis of think-aloud responses and the discussions held at the end of each session are presented in this section in the context of the three key TAM themes used for analysis: PU, PEOU, and AU. A number of sub-themes were identified within these key TAM themes, these are presented as a thematic mind map in Figure 3.

### Perceived Usefulness (PU)

#### Increased Understanding

Participants B, D, E, F, and G felt that 3D images were a good visual aid that enabled patients to have a better understanding of assistive technology or adaptations to be provided. It was felt that the rich visual representations and interactive environment provided by the application is preferable to the static hand-drawn examples that are often used in practice. The 3D images were also seen to somehow convey additional information that otherwise would be difficult to verbalize in their absence.

I think everyone could have a look and it’s much better than having drawings or trying to explain them.

Participant D

Other participants perceived that it can give the patient “immediate feedback” on planned changes (Participants C, G), which is likely to improve shared understanding of proposed adaptations to the home and the extent to which patients and practitioners can engage in a meaningful discussion about a particular scenario. Participant A felt that comprehension and evaluation of items that might be difficult to determine when looking at an aerial or 2D drawing would be much clearer when presented as a 3D representation. This participant also felt that it would be helpful to spot additional issues that would be lost using 2D representations, such as the height of an oven for a wheelchair user.

#### User Involvement

Participants A, E, F, G spoke about the positive impact of client involvement. They suggested that the use of the application in conjunction with the patient would be likely to empower patients and enable them to share the expertise and knowledge that they have about their unique circumstances, how they manage their condition, and how they engage with their home environment.

They are the experts in their situation and so if we can get them to join in with the design process, it makes it easier for everybody.

Participant A

The software was perceived as supporting shared decision making since patients would be more involved within the process (B, E) and understand the rationale behind the suggested adaptations. Hence, participants felt that the visualization afforded by the application would help to foster improved levels of engagement in the PHV process and reduce the levels of ambivalence and anxiety that sometimes surround the process of introducing assistive equipment into the home environment.

Most people are really a bit ambivalent about equipment. Obviously, first it’s a horrible reminder of things going wrong. And usually it’s because a lot of people can’t visualize it.

Participant E

The application was also perceived as a tool that could be used for the independent assessment of technology and enable parents to design environments for their children (Participant A, C, G). In particular, the negotiation process for introducing new equipment into the home can sometimes be extremely resource intensive, requiring numerous visits to the home to explore concerns regarding space requirements and positioning of equipment. Utilization of the VRIDA application was seen to offer a solution that could potentially reduce the time and resources required to come to an agreement on home adaptations.

Because every parent says they don’t have the space, so when you can show you have room for that, it would be so different what you can do without going so many times in his house

Participant G

#### Utility of Software

One perception (Participant D) was that the software would not be suitable for persons with cognitive impairment. It was felt that such patients may find it challenging to make the connection between the virtual representation of the home environment and the home itself. However, some participants believed that the application would be very helpful when carrying out major modifications to the home environment (Participant B, E) and was more effective than the current method of taking photographs (Participant C, D) or paper drawings (Participant A). One of the advantages of the interactive 3D representations would be that the patients could immediately see the proposed changes, without having to worry about the quality or scaling of the photographs or hand drawings that have been used to come to a decision. It was felt that 3D representations were likely to offer peace of mind and foster better quality and more timely collaboration around a more accurate representation of the individual patient’s home setting.

If you can knock something like this up and send it to them, and even better these days when so many people are online, you can create something back at the office and send it through to them and say, right, this is what I think, what do you reckon?

Participant A

### Perceived Ease of Use (PEOU)

#### Learning to Use the Software

All of the participants were able to complete the assigned tasks and vocalized the way they best learned the new tasks. Participants A, F, E, and G stated that they did not like or use the written instructions provided to assist them in learning how to use the application. All reported that they were able to make sense of the key application functions fairly intuitively without any assistance. Both participants F and G explicitly stated that they preferred to learn by playing with the software as opposed to following written instructions. In contrast, participants B, C and D favored the use of the written instructions for guidance and liked their conciseness and the narrative, which enabled them to engage in a simple task before moving on to designing a home environment of their own conception. Only one participant (Participant C) emphasized the overhead of effort required to practice utilizing the software before any significant progress was made.

#### Operating the Software

A number of issues relating to its usability were identified by participants as a result of carrying out the main task. Participants A, B, and F all commented on how they had difficulty picking up or selecting an item of furniture in order to move it to a new position within the room while completing the task. Participants B, D, E, and F stated that the controls were sometimes tricky to operate. Participants B and G felt that the rotation of objects, in particular, should be done with a dedicated button that would move the object in 90 degree turns, similar to how photos are rotated in digital photo viewing applications. Participants A, B, C, D, E, and F experienced issues or confusion over how to apply a wall texture or color; specifically, the software terms of “left side” and “right side” to wall orientation were unclear. Similarly, Participants B, C, and F felt that the default white color for the floors, walls, and objects made it difficult to visually differentiate between them. Participants B, E, and F felt that the mouse controls were too sensitive.

Participants also made numerous suggestions about additional items that should be included in future versions of the furniture library and OT object catalogue. A summary of the additional items of furniture and assistive equipment suggested by participants are presented in Table 3.

**Table 3:** Suggested additional items for OT^a^object library.

Participant(s)	Suggested item(s)
A, E	Ceiling track hoist
A	Drain (shower room)
F	Folding door or “doors that go both ways”
G	Mirror
C	Non-slip mat
D	OT items for bedroom
C	OT items for kitchen
G	OT items for children’s playroom
A, C, D, E, F	Rails in multiple lengths/rotations
A	Ramps (outdoor items)
E	Sash window
A	Wall-hung basin
A, B	Wheelchair turning radius graphic

^a^OT: occupational therapy

### Actual Use (AU) of the Technology

#### Overview

In general, most participants were positive about the use of the software. Some of the comments were: “it’s quite cool”, “My kids would love it”, and “it’s really great”. Some participants were positive about the value that this application could deliver to occupational therapy more generally and across a range of services.

I think it’s doing a great job. I was really impressed. I think that can really help many OTs throughout the country.

Participant D

Two participants perceived that more work needed to be done if maximum benefits of the application were to be realized in practice. However, they noted that the majority of functionality currently offered is useful and that with only minor adaptations to the interface and functionality, the application would be beneficial to use in practice.

I’m sure it’s a case of fine tuning rather than significant changes.

Participant A

#### Look and Feel

Participants B and G felt that the look and feel of the digital home images needed enhancement to enable a client to connect with the 3D images more effectively. They felt that the home environments presented within the application felt slightly artificial in some way and could benefit from being softened or made to look more ‘lived-in’.

I think it’s got potential, but it still feels quite academic, quite sterile.

Some participants made suggestions relating to how the modelled environments could be made to feel more life-like and lived-in. For example, both Participants B and G suggested making simple additions, such as a rubber duck in the bathroom, to help to add a home-like element that was otherwise felt to be missing. Participant B also suggested a towel on the towel rail, bottles of shampoo, blinds/curtains on the windows, and houseplants.

If you have a bath, where is the bottle of shampoo? Because that’s going to make it look like it is someone’s home. It is easier, it opens up the ability to engage with people who maybe need that household.

Participant B

Participants B and F felt that this technology, specifically the use of a computer and mouse, was outdated. They felt that perhaps delivering interventions using a VRIDA application may be better delivered on more mobile types of platform, such as a tablet computer or a laptop.

#### Measurements

Participants emphasized the importance of measurement and having objects in pre-set sizes (Participants A, C). They felt that it is important to ensure that assistive pieces of equipment are modelled to scale within the environment.

Because at the moment you could end up with a design that looks wonderful but you can’t actually achieve it because you have dropped in a bath that is not actually on the market.

Participant A

Indeed, Participants B and D thought that Sweet Home 3D with exact measurements could be a beneficial tool when communicating with assistive equipment installation technicians. They also felt the exact measurements in Sweet Home 3D would give clients a better representation of what they would be receiving and how it would be oriented. Participant A felt that standardized objects should be included and that the ability to resize or stretch objects in Sweet Home 3D may lead to errors, therefore, suggesting that the sizes of objects within the OT object library should be fixed and protected against being resized within the application.

#### Suitability for OTs

There was a view that the software may be suitable for actual use by OTs working with a variety of clinical conditions. Table 4 summarizes the types of clinical conditions for which SweetHome 3D is likely to be usefully applied to.

One participant perceived that if the technology improved, they would “take it on all of my visits” (Participant G). Another participant perceived that if the technology was to be used in practice, then it must be used with a tablet computer (Participant B).

However, one participant worried about the impact on the profession and was concerned that introduction of such technology could potentially result in less OTs being employed within the profession.

But the one thing I would say, this isn’t necessarily criticism, but it’s just whether not – because it’s been so easily done, whether that actually de-skills OTs and actually kind of takes their jobs away. You won’t need them anymore because you’ve got a whiz-bang computer that can do it for you.

Participant E

**Table 4:** Suggested clinical areas.

Participant(s)	Suggested clinical usage
B, F, G	Children/Young Adults
B	Clients that are difficult to engage
B, D	Long-term Conditions
F	Neurology
F	Older Adults
D	Social Services

## Discussion

### Principal Findings

In this study, occupational therapists viewed the VRIDA software as being a potentially important and useful visual aid to facilitate shared understanding and shared decision making about home adaptations with patients. This is particularly valuable given that, to date, insufficient explanation and notification of home adaptations during home visits has resulted in some users feeling dissatisfied with their experience, resulting in equipment abandonment levels in excess of 50% [12,51,52]. Enabling people to stay at home and maintain independence at home can add to an increased sense of control and improved quality of life [53-55]. The OTs that took part in this study did not seem to be concerned about the usability of the software but rather were more interested in the impact of using a VRIDA as a tool to assist in occupational therapy interventions and the positive impact this would have on the patient experience. Interestingly, evidence from a study involving older adults from 11 European countries found that older adults wanted to have a trusting relationship with the practitioners, to have their preferences respected, and to receive clear health information from the health care providers [56]. The use of VRIDAs within the PHV process was seen as having promising potential to improve patient/practitioner communication and collaboration within practice.

OTs perceived that the VRIDAs may also reduce anxiety and empower patients. Therefore, the use of VRIDA is likely to encourage therapists to consider new mechanisms to promote health literacy, which is a key enabling factor for patients to be empowered, take ownership, and be involved in the decisions that are made about their care. Health literacy is defined as the ability to “access, understand, evaluate, and communicate information as a way to promote, maintain, and improve health in various settings over the life-course” [57]. Traditionally, health literacy tools have taken the form of information leaflets and/or delivery of information verbally. Some of the benefits of using information leaflets have been seen to be that patients and service users are able to refer back to written health information when required and to use the information at their own pace [58]. However, a relationship also exists between poor literacy skills, poor health, and poor health outcomes [59,60]. The use of more visually focused health communication tools, such as VRIDAs, are likely to provide the opportunity to overcome some of the communication imbalances that exist in current practice settings. Indeed, one recent study exploring the use of a virtual reality application to assess whether it could be used for persons with intellectual disabilities to achieve improved provision and communication of health-related information has achieved very promising results [61].

VRIDA applications such as SweetHome 3D were perceived by OTs as having the potential to address miscommunications that typically occur as part of the PHV process, as it gives patients immediate visual feedback on proposed home adaptations. Therapists appeared to believe that patients may prefer visual aids to facilitate understanding as opposed to more traditional methods of communication. To date only one study appears to exist within the research literature which explores the use of visual aids, in the form of photographs, within the process of occupational therapy home modifications or provision of assistive technology. Daniel et al [62] used photographs of the patient’s home to evaluate the validity and feasibility of onsite home assessments with fallers. This technique could have significant benefits for persons with literacy issues, particularly as health literacy is more common among older adults [63]. In addition, evidence-based guidelines published by the Gerontological Society of America [64] suggest that visual aids can help address hearing-related communication issues. Moreover, visual aids can reduce the need for complex verbal information and reduce the cognitive effort required to understand information.

All the participants in this study were able to use the software, thus giving support to the notion that the majority of therapists can utilize technology and not just Generation X [65]. An Australian study [66] found that while therapists did use technology in practice, they used it primarily for client contact, professional development, and professional networking rather than for therapy provision. On the whole, participants were positive about the VRIDA software but two participants perceived the application as needing further refinement. In this study, most of the participants identified features that could enhance the software. Some participants viewed it as “quite sterile” and as being in need of further customization in order to achieve a more personalized look and feel, such as adding objects to the modelled environment that had some personal meaning and would make the environment feel more lived in. Indeed, evidence suggests that much of the personal home is tied up with the association of the self and identity [67]. Issues were also associated with exact measurement and whether this could hinder the development and use of the tool. Measurement is an interesting area of practice within occupational therapy, as to date little is known about how therapists measure for assistive technology and/or the instructions that are given in order to carry out measurement tasks [68].

Unlike many VR studies, this research was not tied to a specific clinical context, condition, or desired outcome such as learning surgical reconstruction [69], client interventions concerning public speaking [19], or interventions with stroke patients [70]. However, the wide variety of suggested uses for the different client groups, suggested by the participants, indicates the potential versatility and applicability of VRIDAs such as SweetHome 3D. One therapist did not perceive it as being useful for persons with cognitive issues, although one study has utilized the Engaging Platform for Art Development (ePAD) for persons with dementia in creative occupations with some success [71]. This study showed that a sample of people employed in occupational therapy possessed computer skills and that the software of choice was indeed usable, as also seems to be the case with the participants in this study. While this may seem like a small step, establishing these key points echoes the work of Laver et al [70] and the opinions of Verdonck and Ryan [15]. Much VR research focuses on the patient and their interactions with the software, without mention of whether or not clinical staff can operate it and feel confident doing so.

### Limitations

A limitation of this research is that a follow-up interview was not carried out separately to the trial session itself; however, interviews were carried out at the end of the think-aloud sessions. This provided them with a chance to share any additional comments and reflect on the experience of using the software application. Qi [72] suggested that a follow-up interview may also allow the participants to validate the researchers’ interpretation of their think-aloud utterances. This study relied on the recruitment of participants who were self-motivated to learn how to use the software, which may indicate that they felt comfortable in their own computer skills prior to participating in this research. In order to attract participants with a wide range of computer literacy, invitations could have explicitly stated that participants who have low as well as high computer skills are welcome to take part in the study. However, the insights gained from this study do represent views of OTs who have significant experience across a wide range of clinical settings and application domains. The number of participants that took part in this study may be considered to be too small to make generalizations about OT perceptions of the use of VRIDA in the PHV process more generally. However, in accordance with recent research findings in the usability testing research domain, the number of participants that took part in this study exceeds the suggested threshold number of five participants that are necessary to provide useful and effective feedback when using the think-aloud protocol for interactive prototype evaluation [40]. In relation to the TAM model, it is noted that it has been advocated that the Human Activity Assistive Technology (HAAT) model integrates the social model of disability, concepts from occupational therapy theory, and principles of assistive technology adoption and abandonment. Hence, if HAAT was used in addition to TAM, additional insights relating to these aspects may have been identified [73]. Nevertheless, TAM is a well-recognized model that has been used with significant success to identify barriers to adoption of new technology within health care and a variety of other settings and provided an appropriate framework through which issues relating to the adoption of VRIDA were identified in a considered way.

### Conclusions

This study has gained valuable insights into the value and utility of using VRIDA software applications such as SweetHome 3D within the occupational therapy setting and more specifically within the PHV process. OTs appeared to be positive about the utilization of VRIDAs within a range of clinical settings and that it would serve as a valuable collaborative tool that could empower patients and facilitate more effective patient/practitioner engagement. The study also revealed that VRIDAs have the potential to facilitate decision making and could serve as a valuable tool to demonstrate ideas and put them into a visual context that is personalized and intuitive for the patient. Furthermore, using VRIDA could better facilitate shared decision making and empower patients to play more of a role in the decisions that are made about their care. This is especially important given the complex emotions that can be tied to conditions leading to home modifications or the need for equipment. Furthermore, many studies look at the patient experience without noting the experience from the point of view of the clinician. It is often assumed that clinicians have/do not have the ability to learn to use new technology in practice. Without gathering and documenting the clinician’s perspective, research is missing the valuable insights that clinicians can bring as a result of their range of clinical experience and that can be fed back into the development of technology that is tailored to the clinicians needs. This study has identified a number of issues that now can be addressed in order to ensure that the proposed VRIDA technology is suitably adapted and made to be fit for purpose, if it is to be introduced as a tool to facilitate more effective PHV interventions. Ultimately, new tools and strategies that enable improved patient/practitioner communication and collaboration must be identified and deployed, if significant levels of equipment abandonment seen as a result of PHV interventions are to be addressed and overcome. The use of VRIDA as a tool to facilitate improved communication and collaboration within this process has been perceived to be promising by practitioners.

Further research is needed to explore patient perceptions of VRIDA and to better understand the effectiveness of using such applications jointly and collaboratively with patients and practitioners. Further development work is also needed to incorporate the requirements suggested by practitioners as a result of this study and to identify patient specific requirements, which will ensure that both patients and practitioners are able to optimally benefit from using this application in practice.

## Abbreviations

AU: actual use
HAAT: human activity assistive technology
ICT: information and communication technology
NHS: National Health Service
OT: occupational therapist
PDA: personal digital assistant
PEOU: perceived ease of use
PHV: pre-discharge home visit
PU: perceived usefulness
TAM: technology acceptance model
VR: virtual reality
VRIDA: virtual reality interior design application
